# Relation of Visceral and Subcutaneous Adipose Tissue to Bone Mineral Density in Chinese Women

**DOI:** 10.1155/2013/378632

**Published:** 2013-06-03

**Authors:** Ling Wang, Wei Wang, Li Xu, Xiaoguang Cheng, Yimin Ma, Dan Liu, Zhe Guo, Yongbin Su, Qianqian Wang

**Affiliations:** ^1^Department of Radiology, Beijing Jishuitan Hospital, Beijing 100035, China; ^2^Department of Radiology, Hong Kong University Shenzhen Hospital, Shenzhen 518053, China; ^3^Department of Epidemiology, Beijing Jishuitan Hospital, Beijing 100035, China

## Abstract

The relationship between adipose and bone tissues is still being debated. The purpose of our study was to evaluate whether the distribution and volume of abdomen adipose tissue are correlated to trabecular bone mineral density in the lumbar spine. In this cross-sectional study, 320 Chinese women, being divided into two groups according to age ≥55 years and <55 years, were evaluated with quantitative computed tomography (QCT) of the spine to simultaneously evaluate the average trabecular BMD of L2–L4, VAT, and SAT. Possible covariates of height, weight, age, and comorbidities were considered. In the <55-year-old sample, multiple linear regression analyses indicated that VAT volume was negatively correlated to trabecular BMD (*P* value = 0.0003) and SAT volume had no correlation to trabecular BMD. In contrast, there was no significant correlation between VAT or SAT and BMD in the ≥55-year-old sample. Our results indicate that high VAT volume is associated with low BMD in Chinese women aged <55 years and SAT has no relation with BMD.

## 1. Introduction

Low bone mineral density (BMD) has long been established as an important risk factor for hip fracture or lumbar fracture [1]. It follows that knowledge of the other factors, such as obesity, smoking, alcohol intake, drugs intake, and long-term bed rest, influencing BMD is crucial for preventing and treating osteoporotic disease. Among these factors, obesity was previously thought to have a positive influence on the maintenance of BMD [2–5]. However, recent studies have documented an obesity paradox of lower bone density in obese than normal weight subjects with particular conditions [6, 7]. A number of studies reported the potential physiological mechanisms that may lead to obesity paradoxes [8–10], but the topic is far from being definitively settled.

Little is known regarding the distribution of adipose tissue in terms of visceral (VAT) and subcutaneous (SAT) compartments on bone fragility. Although there is evidence that amount of visceral adipose tissue (VAT) plays a deleterious role in many other diseases [11], there is little information on how VAT and SAT affect bone mineral density [12–14]. Therefore, further studies are needed to explore the possible effects of VAT and SAT on BMD.

To our knowledge, there have been no previous studies on the possible independent effects of VAT and SAT on BMD in Chinese women. The purpose of our study was to investigate whether the distribution of abdomen adipose tissues influences trabecular BMD of the lumbar spine. We chose to use cross-sectional images using quantitative computed tomography to simultaneously estimate the volumes of VAT and SAT as well as vertebral trabecular bone density.

## 2. Materials and Methods

### 2.1. Subjects

Subjects included 320 Chinese women aged 19–86 years having QCT examinations from February 2010 through October 2012. Some subjects had their first QCT examination at our hospital for an assessment of their bone mineral density. The others were recruited from a pool of outpatients for abdomen or Lumbar spine CT scan. The QCT dataset could be achieved with calibration phantom scanned beneath the body simultaneously, without additional radiation and relocation of the patients. These patients completed written informed consent forms before any measurement. The menopausal status could not be confirmed in all subjects, as some of them could not be able to accurately determine when they begin to be menopausal or did not remember accurate age of menopause. An epidemiological survey including 15083 subjects indicates that the mean age at spontaneous menopause was 50.6 ± 3.7 years old in Chinese women [15]. Prior pointed out that the term “perimenopause” could be used to characterize women between the ages 45 and 55, given the lack of clarity about the onset of the perimenopause [16]. So the subjects were divided into two groups according to ages ≥55 and <55. Most of the subjects aged ≥55 may be postmenopausal to further overcome perimenopausal effect. Participants who had used or were using drugs that have an influence on bone metabolism were excluded. The exclusion criteria also included diabetes, thyroid and parathyroid disease, and liver or renal disease. The study was approved by the Ethics Committee of the Beijing Jishuitan Hospital, 4th Clinical College of Peking University.

### 2.2. Anthropometry

Height and weight were measured to the nearest 0.1 cm and 0.1 kg respectively when the subjects wore the underwear. All values were recorded as the mean of two repeated measures. BMI was calculated as the weight (kg) divided by the square of the height (meters).

### 2.3. QCT Measurements of BMD

All subjects underwent cross-sectional CT scan of the abdomen from the level of the second to the fourth lumbar vertebral body (L2–L4) with the same CT scanner (Aquilion, 16 Toshiba, Tokyo, Japan). Scan parameters were 120 kV, 100 mAs, 1 mm slice thickness, and 40 cm field of view (FOV). Trabecular bone mineral density (BMD) measurement of L2–L4 was performed using a software package: QCT PRO 4.2.3 (Mindways, Austin, TX, USA). Subjects were positioned supine on the CT table with the same Mindways CT calibration phantom placed under the subjects to cover levels L2 to L4. Images were transferred to the QCT PRO PC (2007 Mindways Software, Version 4.2.3; Mindways, Austin, TX) by the image transfer utility set up on the CT scanner. A region of interest within trabecular bone of each of three vertebral bodies was placed semiautomatically for the BMD measurement, so as to avoid cortical bone and posterior veins. The average trabecular BMD of L2–L4 was calculated. The precision for this technique is less than 1.5% [17, 18].

### 2.4. QCT Measurements of Adipose Tissue

We measured adipose tissue in L4 level slice. This slice typically intersects the umbilicus and is consistent with other CT protocols for VAT measures. The umbilicus cross-section was chosen because it has the maximum ratio of fat to total tissue area and the visceral fat area at the umbilical region has been found to be strongly correlated with visceral fat volume (*r* = 0.921 in males and 0.931 in females) [19, 20]. On the same CT images, measurements of total adipose area (TAA) and visceral adipose area (VAA) were semi-automatically completed by the commercial software package: “Tissue Composition Module” Beta 1.0 (Mindways, Austin, TX, USA). For the purposes of this study, SAT was defined as the area of adipose tissues between the skin and the rectus muscles of the abdomen, the external oblique muscles, the broadest muscle of the back, and the erector muscles of the spine at the level of L4. VAT was defined as all intra-abdominal adipose tissue area within the abdominal cavity of rectus, external oblique, lumbar quadrate, and psoas muscles. All the measurements were carried out by a single trained in the QCT techniques.

### 2.5. Statistical Analysis

Statview 9.0.1 (SAS Institute Inc., Cary, NC) was used for the statistical analysis. Results are presented as mean ± SD. All variables were checked for outliers and normality using Shapiro-wilk tests. Because all continuous variables were nonnormally distributed, associations among the independent variables were explored using nonparametric Spearman rank correlation coefficients. Multiple linear regression was used to assess the relationships between abdominal adiposity and trabecular BMD. Statistical significance was accepted at *P* < 0.05.

## 3. Results

**Figure 1:**
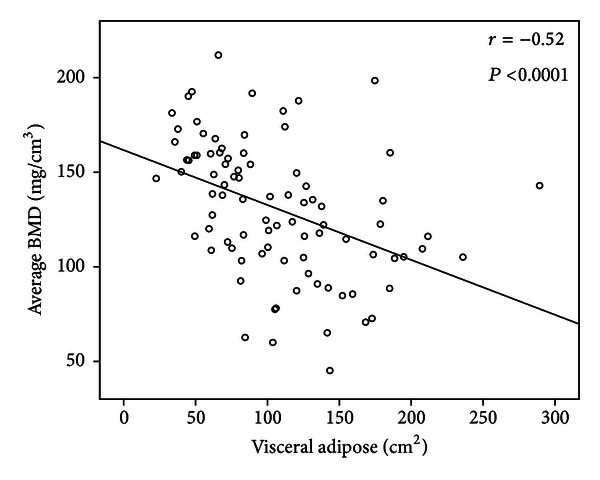
Correlation between average bone mineral density (mg/cm^3^) and visceral adipose tissue in group aged <55. “*r*” is the Spearman correlation coefficient.

**Figure 2:**
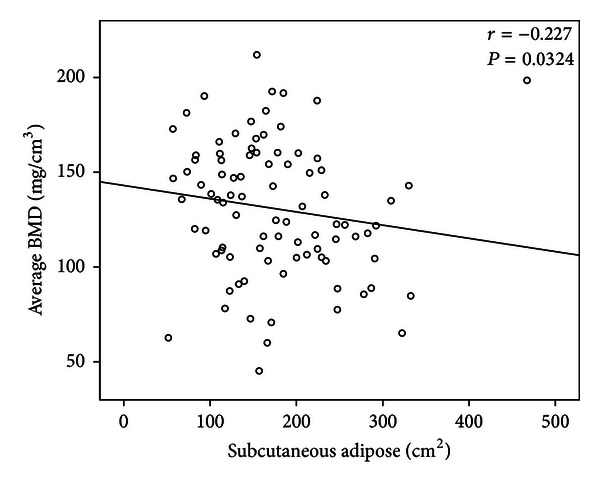
Correlation between average bone mineral density (mg/cm^3^) and subcutaneous adipose tissue in group aged <55. “*r*” is the Spearman correlation coefficient.

The descriptive statistics for the samples are shown in Table 1. The age of young and elder sample ranged from 19 to 54 years and from 55 to 86 years, with a mean and SD of 41.51 ± 10.60 years and 66.1 ± 7.34 years, respectively. In the group aged <55 years, nonparametric Spearman rank correlation coefficient analysis showed average trabecular BMD to be negatively correlated with age and BMI, whereas there was no association between average trabecular BMD and weight (*P* = 0.26) (Table 2). There was an inverse correlation between average trabecular BMD and VAT (*r* = −0.52, *P* < 0.0001) (Figure 1), which remained significant after adjustment for age and BMI using multiple liner regression analysis (*P* = 0.01) (Table 4). TAT was found to be not correlated with trabecular BMD using regression analysis (Tables 2 and 4). Whereas spearman correlation between SAT and average trabecular BMD was negative (Figure 2), multiple regression analysis showed that SAT, after accounting for age and BMI, had no correlation with BMD (*P* = 0.88). In contrast to the significant correlation found in the group aged <55 years, there was no significant association between any adiposity and average trabecular BMD in the group aged ≥55 years (all *P* > 0.05) (Tables 3 and 5).

## 4. Discussion

Our results indicate that VAT may be deleterious to BMD but that SAT appears not to be correlated with BMD in the young Chinese women. There also appears to be no correlation between abdominal adipose tissue and trabecular BMD in the elder Chinese women. More fat accumulation is a known risk factor for cardiovascular disease, hypertension, and diabetes; however, the role of abdominal adiposity on BMD is still being debated. Recent studies suggest the adipose tissue is detrimental to the maintenance of BMD [21–23] and a number of physiological mechanism studies have confirmed this hypothesis [8–10, 24, 25]. Though abdominal adiposity may have a direct effect on skeletal loading and may have a positive effect on BMD [26], the relation between adipose tissue and bone is complicated. Both osteoblasts and adipocytes originate from a common progenitor and bone marrow skeletal stem cells MSC [8], and their differentiation is regulated through the PPAR-*γ* (peroxisome proliferators activated receptor-gamma) pathway. Activation of PPAR-*γ* drives the differentiation of MSC towards adipocytes over osteoblasts [9]. Furthermore, the neuropeptide Y (NPY) system acts to regulate both bone and fat tissue in a coordinated manner, and this remains a strong candidate for mediating interactions between these two tissues [10]. Secretion from adipocytes may have both negative and positive effects on bone [9].

Visceral and subcutaneous adipose tissues express different adipokines. Visceral fat induces an increased risk of cardiovascular and metabolic complications, whereas subcutaneous fat exerts some still undefined protective actions [27]. Our data confirms the hypothesis that the different distributions of abdominal adipose tissue may have different influences on trabecular BMD. A recent study suggests that visceral fat is detrimental to femur structure and strength, whereas subcutaneous fat is beneficial to bone [28]. This suggests the same relationship between VAT and bone that we found in our study. However, it would appear that SAT may have a different relationship with bone compared to our study, although our study involved measurement of the trabecular BMD of lumbar spine and not the femur. It may be that the subcutaneous adipose tissue exerts mechanical stress on bone and therefore acts positively on femur structure and strength. 

Other studies confirmed the negative relationship between VAT and bone [21–23]. Nevertheless, the results of SAT and bone in observational studies are somewhat controversial. Both negative [23, 29] and positive [28, 30] associations between SAT and bone mass have been reported. In our study, the average trabecular BMD is negatively associated with SAT in the group aged <55, whereas no relation was found in regression analysis adjusting for BMI and age (*P* = 0.88). This suggests that the relation between adiposity and BMD may be confounded by BMI and age.

We used QCT to assess the BMD of vertebral body. Most previous studies have assessed bone parameters by DXA. Area bone mineral density is dependent on skeletal size, so it will correlate with any other variable (such as lean mass) which is also dependent on skeletal size. DXA measures area bone density (g/cm^2^) so is also influenced by bone size, as well as the mineral density of the bone being assessed [31]. This limitation leads inevitably to a relationship between body mass and bone mass or areal density. It is therefore important to produce a measurement of bone mineral density that takes account of this problem. This can be done by using QCT, which directly assesses volumetric bone density [26]. Furthermore, fat layering introduces error and decreases the reproducibility of DXA spine and hip BMD measurements in human volunteers. Although overlying fat also affects QCT BMD measurements, the error is smaller and more uniform than with DXA BMD [32]. QCT measures trabecular BMD and true volumetric bone density, irrespective of bone size. So the measurements of QCT may demonstrate a more accurate relationship between adiposity and BMD.

Although the subject populations where divided on an age-related basis, our results indicate that the relationship between adiposity and bone may be different when separating pre- and postmenopausal women. The menopausal transition is associated with substantial bone loss but a gain in fat mass. Numerous cross-sectional studies show an onset of bone loss at the average age of menopause and lack of consistent; normal ovulation is associated with accelerated bone loss [16]. The negative relation between VAT and BMD in the group aged <55 is consistent with previous studies. Katzmarzyk et al. have recently suggested that VAT was negatively associated with BMD in the younger age group (*β* = −0.054; *P* = 0.0001) but not in the older age group (*β* = −0.002; *P* = 0.86) [23]. The reason for this discrepancy between the two aged groups is not clear. Further studies are needed to investigate possible underlying mechanisms.

Our study has several limitations. Firstly, the study is cross-sectional. Secondly, it is not population based and some participants visited the hospital for BMD measurements. Because it is likely that they had lower BMD, associations might have been underestimated. Finally, data on alcohol intake, lifestyle habits, and menopausal age were not available.

## 5. Conclusion

Our results indicate that high VAT volume is associated with low BMD in Chinese women of age <55 and SAT has no relation with BMD, and there also appears to be no correlation between abdominal adipose tissue and trabecular BMD in the elder Chinese women. For the young Chinese women, obesity, especially visceral adipose accumulation, may not only be a risk factor for many diseases but also be detrimental to bone mineral density.

## Figures and Tables

**Table 1:** Descriptive characteristics of the subjects.

	Mean ± SD (range)	
	Age < 55 (*n* = 89)	Age ≥ 55 (*n* = 231)
Age (years)	41.51 ± 10.60 (19.00–54.00)	66.10 ± 7.45 (55.00–86.00)
Height (cm)	162.01 ± 6.83 (150.00–180.00)	160.88 ± 7.30 (140.00–190.00)
Weight (kg)	61.60 ± 11.48 (40.00–100.00)	66.26 ± 12.46 (40.00–110.00)
Average BMD (mg/cm^3^)	130.86 ± 35.56 (45.19–211.95)	66.92 ± 31.63 (4.95–218.34)
BMI (kg/m^2^)	23.45 ± 4.00 (16.60–33.20)	25.54 ± 4.18 (16.02–40.90)
TAT (cm^2^)	280.81 ± 115.10 (79.85–641.84)	357.88 ± 107.05 (67.85–623.44)
VAT (cm^2^)	106.33 ± 51.63 (22.61–289.31)	159.24 ± 54.79 (37.69–326.27)
SAT (cm^2^)	174.48 ± 74.83 (51.70–467.14)	198.64 ± 70.31 (23.60−384.82)

BMD: bone mineral density, BMI: body mass, TAT: total adipose tissue, VAT: visceral adipose tissue, and SAT: subcutaneous adipose tissue.

**Table 2:** Correlations (*r*) between adiposity, age, BMI, and BMD in group aged <55 years.

	Age	Average BMD	BMI	TAT	VAT	SAT
Average BMD	−0.68					
BMI	0.38	−0.22				
TAT	0.53	−0.39	0.67			
VAT	0.69	−0.52	0.59	0.89		
SAT	0.35	−0.23	0.59	0.93	0.67	

All other correlations are significant; *P* < 0.05.

**Table 3:** Correlations (*r*) between adiposity, age, BMI, and BMD in group aged ≥55 years.

	Age	Average BMD	BMI	TAT	VAT	SAT
Average BMD	−0.55					
BMI	−0.12^a^	0.13^d^				
TAT	0.05^b^	0.02^e^	0.56			
VAT	0.18	−0.02^f^	0.43	0.82		
SAT	−0.07^c^	0.05^g^	0.51	0.87	0.46	

All other correlations are significant; *P* < 0.05.

^a^ *P* = 0.06.

^b^ *P* = 0.49.

^c^ *P* = 0.27.

^d^ *P* = 0.06.

^e^ *P* = 0.76.

^f^ *P* = 0.74.

^g^ *P* = 0.43.

**Table 4:** Multiple linear regression including TAT, VAT, and SAT as independent predictors of BMD in group aged <55, adjusting for BMI and age.

	TAT		VAT		SAT	
	*β*	*P*	*β*	*P*	*β*	*P*
Average BMD	−0.04	0.32	−0.22	0.01	0.0079	0.87

**Table 5:** Multiple linear regression including TAT, VAT, and SAT as independent predictors of BMD in group aged ≥55, adjusting for BMI and age.

	TAT		VAT		SAT	
	*β*	*P*	β	*P*	*β*	*P*
Average BMD	0.02	0.35	0.05	0.16	0.008	0.79
